# Supplementary material for: Solubility and Thermal Stability of Thermotoga maritima MreB
Source: Int J Mol Sci. 2022 Dec 16;23(24):16044. doi: 10.3390/ijms232416044 (PMC9785925; doi:10.3390/ijms232416044)
Supplement: Supplementary file 1 [file ijms-23-16044-s001.zip › ijms-2088706-supplementary.pdf]

Supplementary data

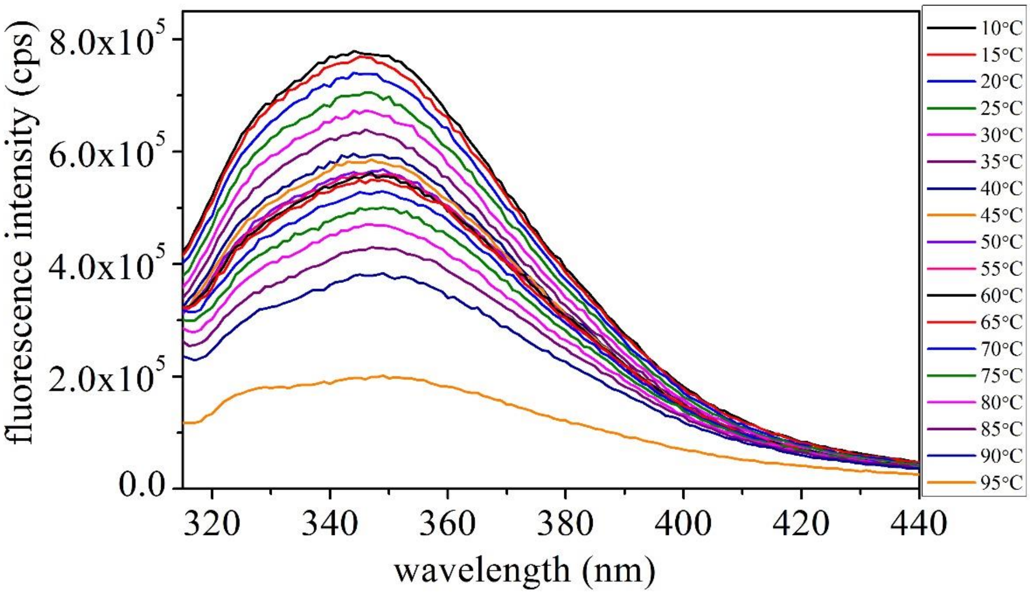

Figure S1. Thermal denaturation of *Tm*-MreB. The fluorescence intensity of tryptophan residue decreases upon temperature increases. At melting point there is a drop in fluorescence intensity change. In case of salt free conditions in presence of 2 mM ATP it can be observed at high temperature, between 90-95°C.

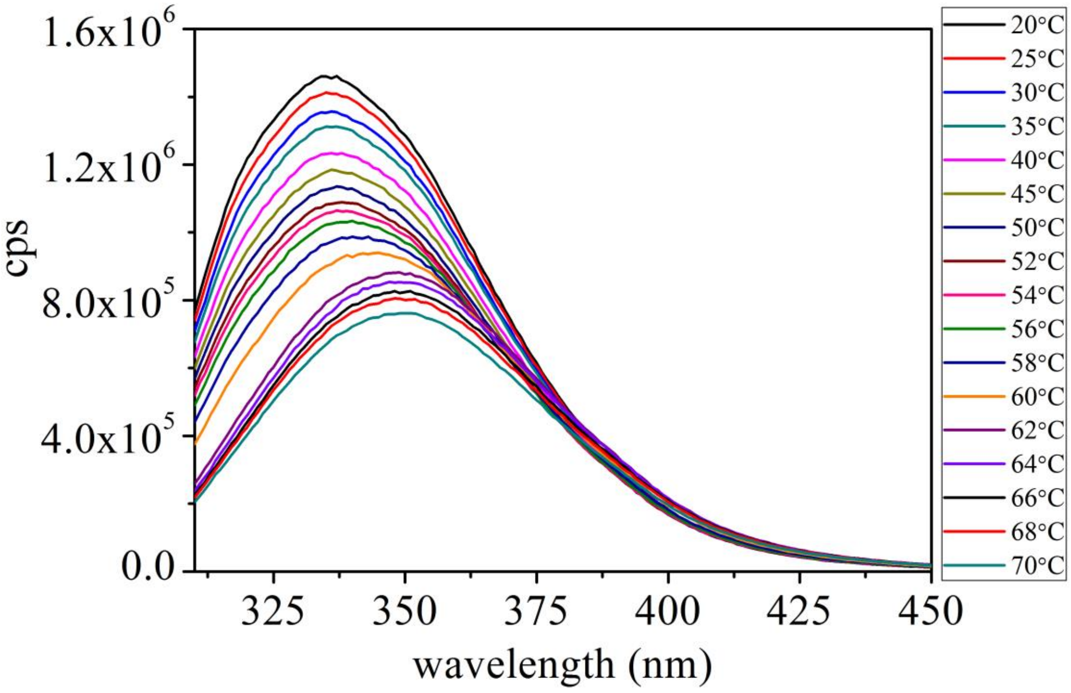

Figure S2. Thermal denaturation of rabbit skeletal alpha actin. Actin was investigated in same buffer than *Tm*-MreB. Beside fluorescence intensity decrease there is also a red-shift observed upon melting.

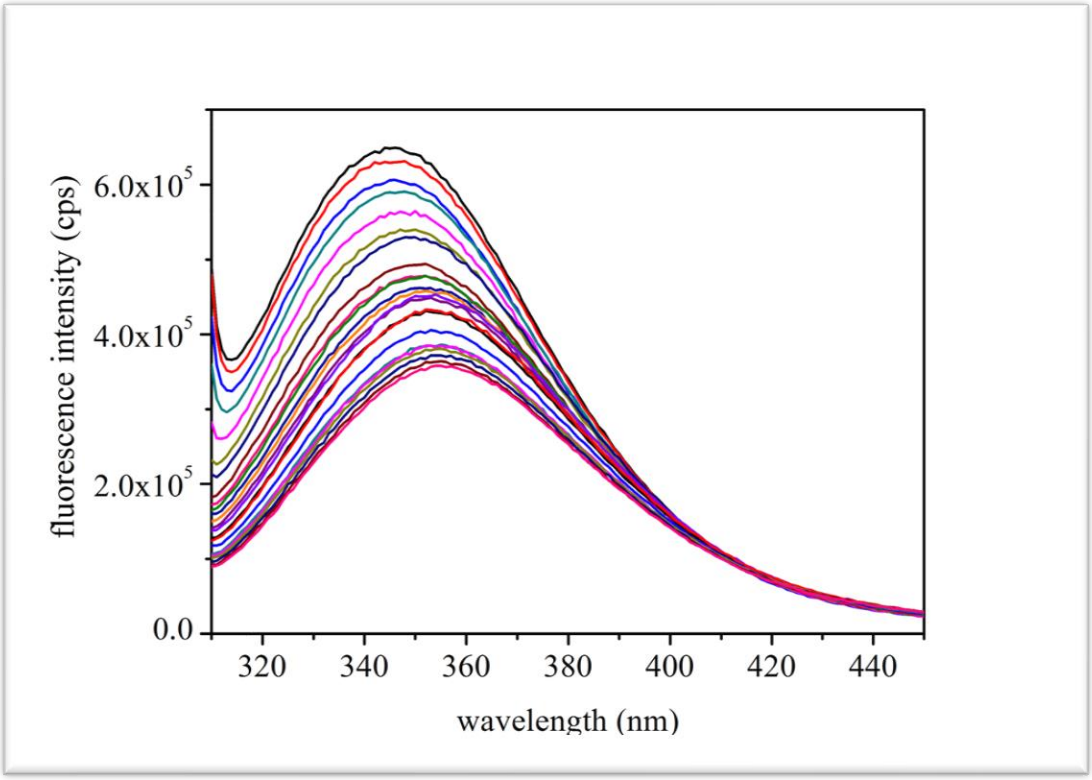

Figure S3. Chemical denaturation of *Tm*-MreB. The fluorescence intensity of tryptophan residue decreases upon concentration of Guanidine hydrochloride increases, and a strong red shift can be observed.
